# Genetic Clues on Implantable Cardioverter-Defibrillator Placement in Young-Age Hypertrophic Cardiomyopathy: A Case Report of Novel *MYH7* Mutation and Literature Review

**DOI:** 10.3389/fcvm.2021.810291

**Published:** 2021-12-23

**Authors:** Xing Li, Jie Tang, Jinhui Li, Sha Lin, Tao Wang, Kaiyu Zhou, Yifei Li, Yimin Hua

**Affiliations:** Key Laboratory of Birth Defects and Related Diseases of Women and Children of Ministry of Education, Department of Pediatrics, West China Second University Hospital, Sichuan University, Chengdu, China

**Keywords:** hypertrophic cardiomyopathy, genetic disorder, *MYH7* mutation, ICD implantation, literature review

## Abstract

**Background:** Hypertrophic cardiomyopathy (HCM) is the second most common cardiomyopathy in childhood with a life-threatening risk. Implantable cardioverter-defibrillator (ICD) placement is recommended for early prevention if there are two or more clinical risk factors. Pediatric patients with HCM are at a higher risk of sudden cardiac death (SCD), but there are limited reports on indications for ICD implantation in children. Herein we describe the case of *Myh7* mutation-induced HCM and cardiac arrest in a patient and evaluated information originating from genetic background to guide ICD administration.

**Case Presentation:** The patient was a girl aged 7 years and 8 months who had been diagnosed with cardiomyopathy *in utero* 8 years prior. She had had recurrent cardiac arrests within the last 4 years. Electrocardiography indicated abnormalities in conduction, and ST segment changes. Echocardiography indicated significant left ventricular hypertrophy and hypertrophic systolic interventricular septum. Cardiac magnetic resonance imaging depicted general heart enlargement with hypertrophy, and delayed enhancement in myocardium with perfusion defect was also evident. Whole exon sequencing identified a *de novo* c.2723T>C (p.L908P) heterozygous mutation in the *MYH7* gene. MYH7 p.L908P predicted unstable protein structure and impaired function. The patient was scheduled for ICD implantation. There were no complications after ICD implantation, and she was discharged from hospital on the 10th day. Regular oral beta-blockers, amiodarone, spironolactone, and enalapril were administered, and she was required to attend hospital regularly for follow-up. During follow-up there were no cardiac arrests. Literature review of clinical prognoses associated with genetic mutations of MYH7, MYBPC3, TNNI3, TNNT2, and TPM1 in pediatric HCM patients with and without ICD implantation indicated that they were totally differently. Previous reports also indicated that gene mutations predicted earlier onset of cardiac hypertrophy, and increase likelihood of SCD.

**Conclusion:** Variant burden and variant type contribute to the risk of adverse events in pediatric HCM. Early recognition and intervention are vital in children. Gene mutation could be considered an indication for early ICD placement during standard risk stratification of HCM patients. Whether this extends to the majority of pediatric patients requires further investigation.

## Introduction

Hypertrophic cardiomyopathy (HCM) is the second most common myocardial disease in childhood, with an annual incidence of ~0.24–0.47 per 100,000. Characterized by unexplained left ventricular hypertrophy (LVH), the typical histological manifestation is uneven distribution of myocardium. The causes of HCM are heterogeneous in children, and include congenital metabolic defects, chromosome-related syndromes, and neuromuscular diseases (1, 2). More than 1,400 rare variants in at least 8 sarcomere-related genes have been linked to HCM, with variations in the *MYH7* and *MYBPC3* genes the most common. Most cases of child-onset HCM are familial/hereditary however, and are caused by mutations in genes encoding the sarcomere, cytoskeleton, or desmosome protein (3).

Children with HCM are at greater risk of sudden cardiac death (SCD) and other adverse events (4). Both the European Society of Cardiology and the American Heart Association recommend implantable cardioverter defibrillators (ICDs) for early prevention if there are two or more clinical risk factors, including severe LVH, unexplained syncope, cardiac arrest, non-sustained ventricular tachycardia, and a family history of SCD (5, 6). However, there is no practical guideline for pediatric patients. Moreover, there is no genetic factor involved in analyzing the risks of pediatric HCM patients suffering SCD. Previous studies indicate that patients with gene mutations are more likely to experience poor outcomes (7). Herein we describe the case of a patient with *de novo MYH7* mutation-induced HCM and recurrent cardiac arrest, then present related literature to assess the influence of genetic risk on early ICD intervention in pediatric HCM.

## Case Presentation

### Ethical Compliance

This report was approved by the Ethics Committee of the West China Second Hospital of Sichuan University (approval number 2014-034). Informed consent was obtained from the patient's parents prior to performing whole exon sequencing, and for the inclusion of the patient's clinical and imaging details in subsequent publications.

### Medical History and Physical Examination

The patient was a girl aged 7 years and 8 months who had been diagnosed with cardiomyopathy *in utero* 8 years prior. She had had recurrent cardiac arrest attacks within the last 4 years (from 2015 to 2021). On each of these occasions she had been saved *via* immediate chest compressions and other cardiopulmonary resuscitation methods. Seizures had been observed two or three times per year, which were ~2–3 min in duration. Her daily activity tolerance was limited, which indicated a NYHA II to III level of heart function. Her parents had no relevant clinical symptoms and were unaware of any family history of cardiovascular diseases or SCD. At rest the patient's ventricular rate was 100–110 beats/min, her breathing rate was 25–35 breaths/min, and her blood pressure was ~95/55 mmHg. Arrhythmia and dull heart sound were detected. Pulmonary, abdominal, musculoskeletal system, and neurological examination results were unremarkable.

### Imaging and Laboratory Examinations

**Figure 1:**
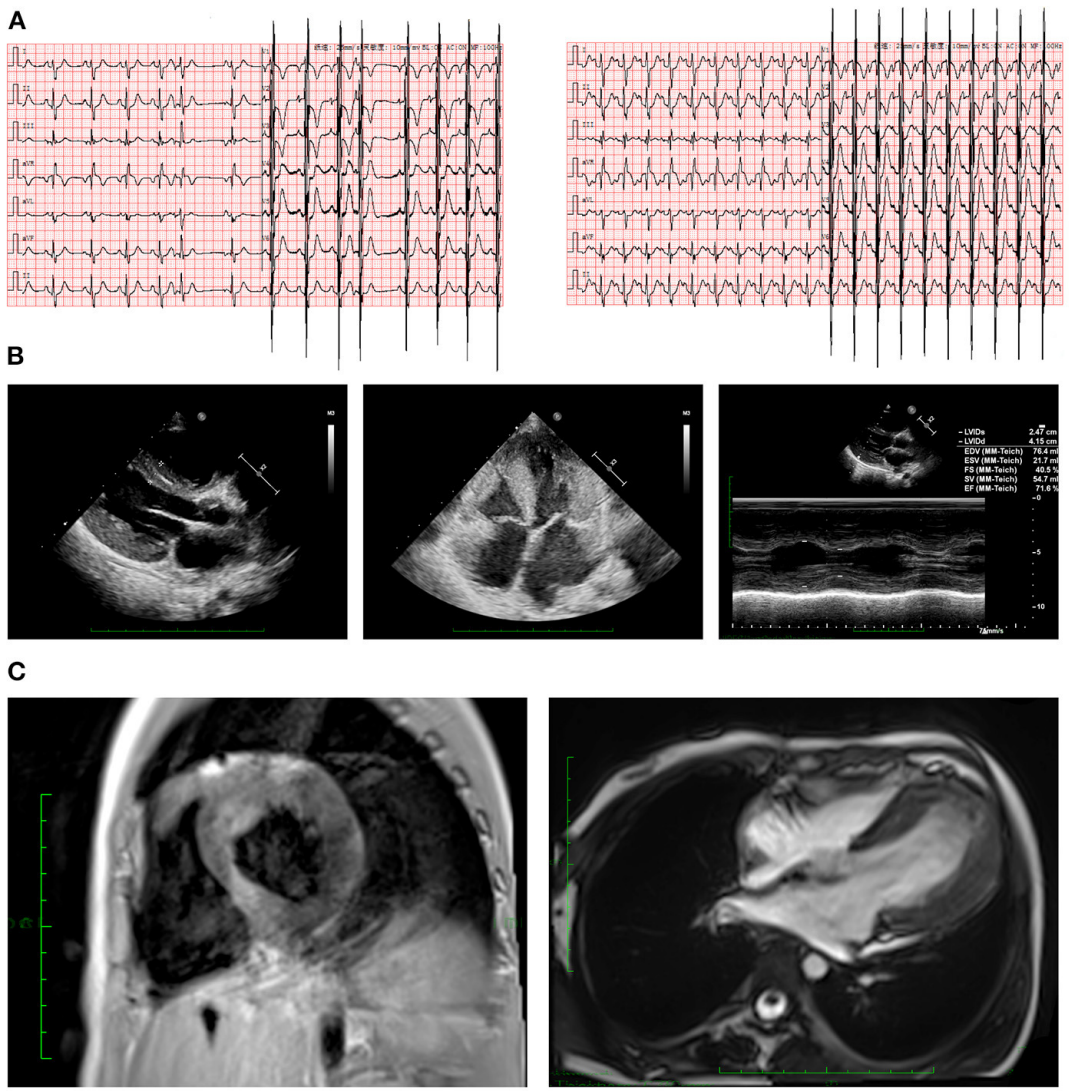
Clinical manifestations in ECG and imaging. **(A)** ECG revealed CRBBB, abnormal double atrium, ventricular hypertrophy, first-degree atrioventricular block, and ST segment changes (lead I, aVL, and V3–V6 depression >0.05 mV). **(B)** Echocardiography indicated severe LVH. **(C)** Cardiac magnetic resonance imaging depicted hypertrophic left ventricular wall in both short axis and long axis views. CRBBB, complete right bundle branch block; ECG, electrocardiogram; LVH, left ventricular hypertrophy.

Electrocardiography (ECG) revealed electrical axis deviation to the right, complete right bundle branch block (CRBBB), abnormal double atrium, ventricular hypertrophy, first-degree atrioventricular block, ST segment changes (lead I, aVL, and V3–V6 depression >0.05 mV) (Figure 1A). Notably however, ventricular tachycardia had been observed three times on a monitor and had failed to be recorded before electric cardioversion. Echocardiography have been performed by the same echocardiographist to make the data comparable, and the echocardiography pre-ICD implantation indicated significant LVH and hypertrophic interventricular septum (19 mm, Z-score = 5.64) at the end of diastole with normal left ventricular systolic function (62–71.6%) (Figure 1B). Cardiac magnetic resonance imaging demonstrated general heart enlargement with LVH. Delayed enhancement in the myocardium with perfusion defect were also detected, indicating microdiffusion disorder in myocardial tissue. Scattered flaky enhancement foci were evident in the myocardium, suggesting myocardial fibrosis (Figure 1C). Initial laboratory tests revealed dramatic elevation of troponin I (> 50 ug/L, n.v. <0.02 μg/L) and B-type natriuretic peptide (10,330.6 pg/mL, n.v. <146 pg/mL) immediately after her first cardiac arrest at age 6. During follow-up before ICD implantation, the troponin I range was 0.15–20.32 ug/L and the B-type natriuretic peptide range was 478.2–8746.1 pg/mL.

### Molecular Results

A peripheral blood sample was obtained from the patient in an ethylenediaminetetraacetic acid anticoagulant blood sample tube then stored at 4°C for <6 h. DNA was extracted using the Blood Genome Column Medium Extraction Kit (Tiangen Biotech, Beijing, China) in accordance with the manufacturer's instructions. Protein-coding exome enrichment was performed using the xGen Exome Research Panel v.1.0, comprising 429,826 individually synthesized and quality-controlled probes targeting 49.11 Mb of protein-coding regions (> 23,000 genes) of the human genome. Whole exon sequencing was performed using the NovaSeq 6000 platform (Illumina, San Diego, CA, USA), and the raw data were processed using FastP to remove adapters and filter out low-quality reads. Paired-end reads were aligned to the Ensembl GRCh38/hg38 reference genome using the Burrows–Wheeler Aligner. Variant annotation was performed in accordance with database-sourced minor allele frequencies (MAFs) and practical guidelines on pathogenicity issued by the American College of Medical Genetics. The annotation of MAFs was performed based on the 1000 Genomes, dbSNP, ESP, ExAC, Provean, Sift, Polypen2_hdiv, Polypen2_hvar, and Chigene in-house MAF databases using R software (R Foundation for Statistical Computing, Vienna, Austria). The sequencing data have been deposited in the GSA database (http://ngdc.cncb.ac.cn/gsub/).

**Figure 2:**
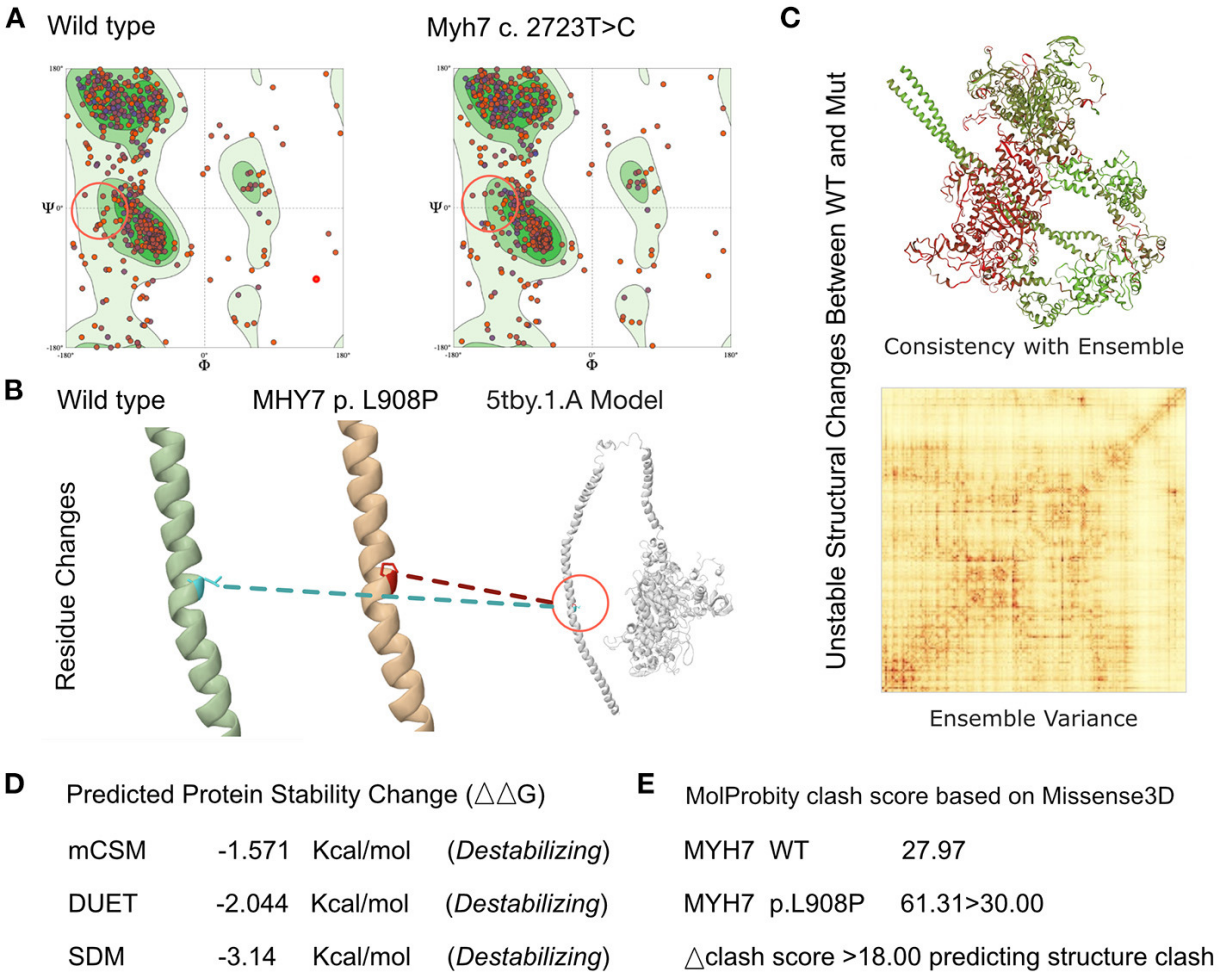
Effects of *MYH7* c.2723T>C mutation on molecular protein structure. **(A)** Ramachandran plots of MYH7 with and without p.L908P mutation. **(B)** SWISS-MODEL to predict the variant's wild-type and mutated protein crystal structures using 5tby.1.A template, and structural changes identified in the helix. **(C,D)** The mCSM tool used to predict protein stability revealed a decline in Gibbs free energy, indicating an unstable protein structure associated with this mutation. **(E)** Missense 3D analysis indicated that MYH7 p.L908P caused a structure clash.

MutationTaster was used with R software to predict the pathogenicity of *MYH7* c.2723T>C and assess the effects of this mutations on protein structure. Besides, according to the recent report from Lioncino et al., we also evaluated the related mutations of RASopathies, and the result was negative (8). There is no MYH7 protein crystal structure available. Modeling analysis was performed using SWISS-MODEL (https://swissmodel.expasy.org/) for the three domains with a 5tby.1.A template. The capability of the protein structure was estimated using Ramachandran plots (Figure 2A). Change in the free energy of the model was estimated using the mutation cut-off scanning matrix (mCSM) method (http://biosig.unimelb.edu.au/mcsm/). Site Directed Mutator (SDM, http://marid.bioc.cam.ac.uk/sdm2) was used to enable assessment of the effects of mutations on the stability of MYH7, and the DUET server was also used (http://biosig.unimelb.edu.au/duet/) to integrate mCSM and SDM to improve the overall prediction accuracy of the mutations under consideration. The signature vector that was ultimately generated was used to train the predictive classification and regression model for calculating the change in Gibbs folding free energy induced by the mutations. Missense3D (http://missense3d.bc.ic.ac.uk/) was used to visualize the structure of amino acid residue changes (wild-type and L908P variant). Clash scores were obtained *via* Missense3D to measure the stability of mutated proteins.

Based on laboratory analyses and the patient's clinical manifestations a genetic disorder was strongly suspected. Whole exon sequencing was performed using the Illumina NovaSeq 6000 platform, and a *de novo* c.2723T>C (p.L908P) heterozygous mutation was identified in the *MYH7* gene. Neither the patient's mother nor her father had this mutation. According to the American College of Medical Genetics these variants have uncertain pathogenicity (PM1+PM2+PM3). *MYH7* c.2327T>C has not been reported in any populations; this is the first report of this variant. Analysis performed with MutationTaster revealed that this mutation is considered pathogenic due to amino acid sequence changes, protein features affected, and loss of helix superstructure (probability = 1 for c.2327T>C). PolyPhen 2.0 predicted this mutation of p.L908P to be “probably damaging” (score = 1.0, sensitivity 0.00, specificity 1.00). The SWISS-MODEL tool was used to analyze stability after amino acid changes. Ramachandran plots indicated that amino acid positions were altered (Figure 2A). Rebuilding molecular structure based on a 5tby.1.A templet resulted in residue changes between Leu and Pro at 908 (Figure 2B). Using SWISS-MODEL protein stability prediction tools, ensemble changes among all the coded amino acids exhibited significant variance (Figure 2C). Three types of calculation methods all demonstrated significant destabilizing change (mCSM, −1.571 Kcal/mol; DUET, −2.044 Kcal/mol; SDM −3.140 Kcal/mol; Figure 2D). The Missense3D tool was also used to evaluate the molecular probity clash score. MYH7 p.L908P predicted obvious protein clash, with an elevated clash score > 18.00 (Figure 2E).

### Final Diagnosis and Treatment

After several examinations and molecular tests the child was diagnosed with HCM, ventricular tachycardia, and Stokes-Adams syndrome. Before ICD implantation she received captopril, beta-blockers, and amiodarone to control heart rhythm. Clopidogrel, spironolactone, and vitamin C were provided to improve heart function. Electrical cardioversion has been used to terminate ventricular tachycardia. In accordance with 2020 American College of Cardiology/American Heart Association HCM guidelines (9) the patient was scheduled for ICD placement after the diagnosis of HCM with the novel *MYH7* mutation c.2327C>T, suffering several cardiac arrests accompanied by sustained ventricular tachycardia, and echocardiography demonstrating an interventricular septum thickness > 15 mm.

After ICD implantation the patient suffered no complications and was discharged from hospital on the 10th day. Oral beta-blockers, amiodarone, and enalapril were then administered for regularity, and spironolactone had been provided at the 1st month post ICD implantation. in conjunction with The patient had been required to visit hospital strictly for follow-up. During the 1 year of follow-up period there was no recurrent cardiac arrest, and the ICD remained functional. The most recent ECG indicated sinus rhythm, CRBBB, LVH, and ST segment changes, and prolonged QT and QTc intervals. During the follow-up, Holters had been performed every 2 months, which were absence of ventricular tachycardia. Echocardiography indicated general hypertrophic changes in left and right ventricular hypertrophy at the end of diastole (interventricular septum 14 mm (Z-score = 4.22), left ventricular posterior wall 9 mm (Z score = 2.90), right ventricular posterior wall 6 mm) and normal left ventricular systolic function = 64%.

## Discussion

HCM is the most common type of cardiomyopathy in children. It is associated with SCD and other adverse outcomes that are usually life-threatening such as severe heart failure, which lead to early death. A recent statement from Monda et al. demonstrated the HCM was the major cause of SCD among all kinds of cardiomyopathies (10). Besides, this statement emphasized the importance of genetic variants evaluation in preventing future SCD. Notably however, predicting such cardiac events in HCM patients remains challenging. Furthermore, the European Society of Cardiology and the American Heart Association developed a risk score system to guide the clinical management of HCM patients. Unfortunately there are no specific recommendations for preventing SCD in pediatric HCM patients, and no specific recommendations pertaining to ICD implantation in such patients. In view of this limitation several case series and reports have discussed this issue and suggested reasonable indications for ICD placement. Because the technique for ICD placement in pediatric patients is challenging and high-risk, the procedure involves several external considerations that do not apply in adults.

Next generation sequencing tests are rapid, and are widely used in both developed and developing countries to investigate genetic associations. Mathew et al. (5) reported that the primary genes affected were *MYH7* in 35% and *MYBPC3* in 49% in a pediatric cohort. *MYH7* mutation-positive patients exhibited earlier disease onset and were at a higher risk of major adverse cardiac events (HR, 2.7, 95% CI 1.3–5.7). The risk of major adverse cardiac events was also higher in patients with multiple variants (*n* = 16; HR, 2.5, 95% CI 1.1–5.9), and *de novo* variant subsets revealed a higher risk (HR, 5.7, 95% CI 2.6–12.7). Ferradini et al. (11) reported that *MYH7* was the dominant genetic site overlapping HCM and arrhythmogenic cardiomyopathy. To predict the risk of SCD among HCM patients, several predictive models had been reported. Recently, two groups reported two individual validated model of SCD in children. The research from Norrish et al. demonstrated the maximal wall thickness and left atrium as the most important indicator to predict 5-years risk of SCD (12). However, this article did not take pathogenic variant into the formula of risk calculation, and they found the family history was not related to the risk of SCD (12). Another research from Miron et al. revealed a validated model for SCD risk. In this model, it confirmed the pathogenic variant elevated the likelihood of experiencing SCD events (HR, 1.32 [95% CI, 1.00–2.12]) (7). Among the reported pathogenic variants, *MYH7* and *MYBPC3* were the most common mutations had been elucidated (7). So that, more detail evaluation of genetic variants, including the molecular function of mutated genes (structural proteins, metabolic molecules or calcium handling channel, etc.) should be taken into different or hierarchical considerations to predict risk of SCD and guiding the application of ICD implantation. This research highlight the importance of genetic analyses and significance of mutations in guiding the implantation of ICD. However, the implantation of ICD among high risk (>6%) population to save patients from SCD was not very satisfied, as only 1 patient may potentially be saved from SCD at 5 years among every 10 ICDs implanted in patients with 6% or more of a 5-year SCD risk (12). So that, the clinical outcomes of ICD application is mainly relied on the patient risk stratification. Analysis of the role of sarcomere genes in the pathogenesis of arrhythmogenic cardiomyopathy is recommended in arrhythmogenic cardiomyopathy patients who test negative for desmosomal mutations. In other research utilizing the International Sarcomeric Human Cardiomyopathy Registry (13, 14) the top three genetic variants indicating HCM onset were *MYH7, MYBPC3*, and *Thin Filament*. The average age at initial diagnosis was 40.1 ± 15.2 years however, thus corresponding pediatric information remains lacking. That research demonstrated that new-onset atrial fibrillation developed in 19% of HCM patients with sarcomere mutations, and compared with other sarcomere genes, patients with pathogenic or likely pathogenic variation in *MYH7* had a higher rate of incident atrial fibrillation independent of clinical and echocardiographic factors. Accordingly, *MYH7* mutation is a major indicator of arrhythmia in HCM patients, which called positive recommendation for ICD application. Besides, the mutations of above genes, a newest research from Lioncino et al. demonstrated the RASopathies was critical to underline the clinical outcomes HCM (8). So that, any HCM patients, especially for the children who had right ventricular hypertrophy involved, should be paid more attention to exclude RASopathies.

In recent years ICD interventions have proven effective for the prevention of cardiac arrest by monitoring and terminating ventricular fibrillation or malignant ventricular tachycardia. ICD therapy in children is associated with higher rates of complications than it is in adults however, necessitating complex consideration before proceeding with ICD implantation (7, 15, 16). Herein we attempted to evaluate the potential benefits of ICD implantation in pediatric patients. Literature review indicated that clinical prognoses associated with genetic mutations (*MYH7, MYBPC3, TNNI3, TNNT2*, and *TPM1*) in pediatric HCM patients with and without ICD implantation were totally different (17–20). Multiple studies indicate that in adults some gene mutations predict earlier disease onset and more severe phenotypes in HCM (21–25). Although gene mutations and phenotypes are heterogeneously influenced by modifier genes, environmental factors, and other influences (16), current evidence suggests that HCM patients with multiple variants are more likely to suffer malignant outcomes and SCD, potentially justifying early ICD implantation (26–29).

Systematic review indicates that ICD implantation is usually recommended in HCM patients with severe cardiac events. Notably however, several large cohort studies and meta-analyses suggest that the most common age range for receiving ICD implantation is ~40–45 years. The current case of HCM was relatively rare in that the patient presented with severe arrhythmic attacks resulting in recurrent cardiac arrest. It was fortunate that death had not occurred prior to ICD placement. ICD placement was deemed urgent in this very young patient (aged 7.7 years). Given her very young age, we acquired all published reports detailing cases of ICD implantation in patients aged under 18 years that provided clear clinical and molecular results. This amounted to a total of seven reports describing 14 cases of patients suffering malignant cardiac events, and almost all of them involved a positive family history. Among them, two patients with compound MYBPC3 variants died soon after birth due to aggressive heart failure with HCM. Another 11 patients presented HCM phenotypes with MYH7, ALPK3, MYBPC3, TNNI3, TNNT2, and TPM1 variants. Age at ICD implantation ranged from 8.5 to 17.0 years. Unfortunately, SCD occurred in 5 of 5 children in whom the ICD placement procedure was not completed. Conversely, only 1 of 7 children (14.3%) died after successful ICD implantation (Table 1). So that, compared with the reports from Norrish et al., which revealed the ICD could only save 1 out of 10 HCM with high risk of SCD without genetic stratification, the patients with positive genetic variant might receive more benefits from ICD implantation according to literature review.

**Table 1:** Association between gene mutations and malignant events in children of hypertrophic cardiomyopathy.

**References**	**Targeted gene**	**Variants**	**Events**	**Age on onset**	**ICD implantation**	**Age on receiving ICD**	**Gender**	**Family history**	**ECG**	**Clinical outcomes**
Fernlund et al. (18)	MYH7	c.746G>A; p.R249Q	Cardiac arrest	7 years	Yes	12 years	Female	Positive	High risk by ECG-risk score	Alive
	ALPK3	c.903del, p.I301Mfs*10	Cardiac arrest	14 years	Yes	24 years	Male	Positive	Several episodes with non-sustained VT	Alive
Hwang et al. (24)	MYH7	c.2146G>A p.G716R	Sudden cardiac death	Young ages	No	N.A.	2 males and 1 female	Positive	Q wave in lead II and aVF	Ceased
Jeschke et al. (20)	MYH7	c.2155C>T p.R719W	Cardiac arrest	6.5 years	YES	8.5 years	Male	Negative	Severe arrhythmia including VT	Alive
Lekanne Deprez et al. (19)	MYBPC3	c.2373_2374insG (maternally) and c.1624+1G>A (paternally)	Cardiac failure	3 days	No	N.A	Female	Positive	First degree AVB, with deep QS complexes in the left precordial leads associated with T wave abnormalities	Ceased
	MYBPC3	c.3288delA; p.E1096fs*92 (maternally) and c.2827C>T; p.Arg943X (Paternally)	Cardiac failure	2 weeks	No	N.A	Male	Positive	Giant P waves, hardly any left sided activity in the precordial leads, and flattened T waves	Ceased
Maron et al. (26)	TNNI3	p.R162W (Homo)	Cardiac arrest	17 years	Yes	17 years	Female	Positive	Diffuse flattened T	Alive
Mori et al. (28)	Compound mutations with MYBPC3 and MYH7	MYBPC3 .472G>A; p.V158M; MYH7 c.788T>C; p.V320M	Sudden cardiac death	Childhood	No	N.A	6 children	N.A	N.A	Ceased
Van Driest et al. (17)	TPM1	c.610T>C; p.L185R	Cardiac arrest	2.5/8/10 years	2 cases received ICD	2 years after diagnosis	2 Males and 1 female	Positive	Ventricular fibrillation	1 ceased case without ICD implantation
Monda et al. (30)	TNNT2	c.304C>T; p.A102T	Cardiac arrest	11 years	Yes	N.A.	Male	Positive	N.A	Alive

*ICD, implanted cardioverter defibrillator; N.A., not available; VT, ventricular tachycardia*.

The present case and the associated literature review facilitated the derivation of the genetic clues for a potential future perspective for ICD placement, as the patients with genetic mutations associated with HCM and arrhythmia, especially *MYH7* and *MYBPC3* mutations might be benefit from early ICD implantation. Besides the screening for positive genetic variant, several indicators required to be clarified to demonstrate the risk stratification for SCD. The echocardiographic parameters, especially for left atrium and right ventricle; the onset age with or without syncope; and whether there were notable abnormal ECG changes. However, two studies provided a controversial results on the application of ECG in predicting SCD (31, 32). So further prospective studies are required to provide more evidences on SCD application indication among HCM children.

## Conclusion

Variant burden and variant type contribute to the risk of adverse events in pediatric HCM patients. Early recognition and intervention are vital in such patients. We suggest that gene mutation could be considered as an potential indication for early ICD placement in pediatric HCM patients during standard risk stratification, but this requires further investigation.

## Data Availability Statement

The datasets presented in this study can be found in online repositories. The names of the repository/repositories and accession number(s) can be found at: http://ngdc.cncb.ac.cn/gsub, GSA database.

## Ethics Statement

The studies involving human participants were reviewed and approved by the Ethics Committee of West China Second Hospital of Sichuan University (2014-034). And informed consent from the patient's parents prior to conducting the WES had been obtained, including the patient's clinical and imaging details in the manuscript for the purpose of publication. Written informed consent to participate in this study was provided by the participants' legal guardian/next of kin. Written informed consent was obtained from the individual(s), and minor(s)' legal guardian/next of kin, for the publication of any potentially identifiable images or data included in this article.

## Author Contributions

YH, KZ, and TW were the patient's physicians. XL and JT reviewed the literature and contributed to manuscript drafting. JL and SL performed the mutation analysis. YL conceptualized and designed the study, coordinated and supervised data collection, and critically reviewed the manuscript for important intellectual content. YH and YL were responsible for the revision of the manuscript for important intellectual content. All authors issued final approval for the version to be submitted.

## Funding

This work was supported by grants from the National Natural Science Foundation of China (No. 81700360), Technology Project of Sichuan Province of China (2020YFS0101 and 2020YFS0102).

## Conflict of Interest

The authors declare that the research was conducted in the absence of any commercial or financial relationships that could be construed as a potential conflict of interest.

## Publisher's Note

All claims expressed in this article are solely those of the authors and do not necessarily represent those of their affiliated organizations, or those of the publisher, the editors and the reviewers. Any product that may be evaluated in this article, or claim that may be made by its manufacturer, is not guaranteed or endorsed by the publisher.
